# High Accuracy Acoustic Relative Humidity Measurement in Duct Flow with Air

**DOI:** 10.3390/s100807421

**Published:** 2010-08-09

**Authors:** Wilhelm van Schaik, Mart Grooten, Twan Wernaart, Cees van der Geld

**Affiliations:** 1 Van Schaik Innovation Handling B.V., Glaslaan 2, Building SWA 1.032, 5616 LW, Eindhoven, The Netherlands, E-Mail: wvschaik@humitemp.com; 2 Process Technology, Department of Mechanical Engineering, Eindhoven University of Technology, Den Dolech 2, 5600 MB, Eindhoven, The Netherlands

**Keywords:** acoustics, relative humidity, humid air flow

## Abstract

An acoustic relative humidity sensor for air-steam mixtures in duct flow is designed and tested. Theory, construction, calibration, considerations on dynamic response and results are presented. The measurement device is capable of measuring line averaged values of gas velocity, temperature and relative humidity (*RH*) instantaneously, by applying two ultrasonic transducers and an array of four temperature sensors. Measurement ranges are: gas velocity of 0–12 *m*/*s* with an error of ±0.13 *m*/*s*, temperature 0–100 °C with an error of ±0.07 °C and relative humidity 0–100% with accuracy better than 2 % *RH* above 50 °C. Main advantage over conventional humidity sensors is the high sensitivity at high RH at temperatures exceeding 50 °C, with accuracy increasing with increasing temperature. The sensors are non-intrusive and resist highly humid environments.

## 1. Introduction

Relative humidity is an important parameter that determines product quality and process economics in many industrial processes [1]. Some typical fields of applications are industrial drying, chemical and pharmaceutical industry, production of plastics, flue gas measurement in power plants, agriculture, food processing, heating, ventilation and air conditioning, paper production and coloring of textiles.

One option to measure relative humidity is by means of acoustic techniques. From kinetic gas theory it follows that the speed of sound in air depends on the composition and condition of the air [2]. The most important parameters that determine the speed of sound in air are: temperature, relative humidity, *CO*_2_ concentration and to a lesser extent absolute pressure. By simultaneous measurement of speed of sound and air temperature, relative humidity can be calculated for given pressure and *CO*_2_ concentration.

Acoustic sensors are non-intrusive, in contrast to other conventional humidity sensors like wet and dry bulb sensors or capacitive sensors. This ensures no pressure loss for in-line applications, high life expectancy and insensitivity to contamination. Another advantage over conventional sensors is the high temperature range. Most important advantage is the high sensitivity and the increase of sensitivity with increasing temperature.

Recently, many studies for humidity sensors have been published, particularly acoustic sensors. However, most of these recent developments in acoustic humidity sensors are based on Surface Acoustic Wave (SAW) sensors, which works with another principle, see the work of Wu *et al.* [3], for example. A comprehensive review on magnetoelastic sensors which can be applied for humidity measurements is given by Grimes *et al.* [4]. The above mentioned conventional and SAW techniques for humidity measurement are local techniques, *i.e.*, with a measuring volume in the order of one cubic *mm*. The acoustic technique of the present paper, on the other hand, yields a chordal beam average of humidity in the desired portion of the duct. Another type of acoustic sensor was developed by Zipser *et al.* [5], which has a different layout and is not in-line. Tsai *et al.* uses an ultrasonic sensor for temperature measurement with a correction for humidity [6].

In the present study, the design and tests of a high accuracy in-line acoustic relative humidity sensor for flowing air-steam mixtures in a duct flow are presented. This includes theory, construction, calibration, considerations on dynamic response and results.

## 2. Theory

The speed of sound in a gas for which the second virial coefficient, *B*, is given, can be calculated from [2,7]. In the equation below, *T* is in *K*:

(1) $$c_{0}^{2}=\gamma \frac{\mathrm{RT}}{M}\left(1+\frac{2\mathrm{pB}}{\mathrm{RT}}\right)$$

For each constituent of a gas mixture, *γ* and *B* must be known to calculate the speed of sound in the gas mixture. By measuring the speed of sound at constant temperature, *T*, and pressure, *p*, determined from measurements of air, the composition of air at constant *T* and *p* uniquely depends on the speed of sound.

The constituents of standard dry air according to ISO norm 2533 are listed in Table 1.

Only the concentrations of *N*_2_, *O*_2_, *Ar*, *CO*_2_ and *Ne* and the amount of water vapor have a significant effect on the molar mass of air. If the composition is assumed to be constant except for the amount of water vapor, the mole fraction of water can be determined from the speed of sound.

The use of the second virial coefficient *B* of a mixture of gases to calculate humidity, *RH*, is examined in [2]. Much more convenient to use is the following approximate equation:

(2) $$\begin{array}{r} c\left(T,p,x_{w},x_{c}\right)=a_{0}+a_{1}T+a_{2}T^{2}+\left(a_{3}+a_{4}T+a_{5}T^{2}\right)x_{w}\\ +\left(a_{6}+a_{7}T+a_{8}T^{2}\right)p\\ +\left(a_{9}+a_{10}T+a_{11}T^{2}\right)x_{c}\\ +a_{12}x_{w}^{2}+a_{13}p^{2}+a_{14}x_{c}^{2}+a_{15}x_{w}px_{c} \end{array}$$

The coefficients {*a_i_*} are determined by calibration in reference air of known temperature, *T*, known humidity, *RH* and known speed of sound at the measurement frequency, *c*. From 2, the mole fraction of water vapor, *x_w_*, is determined. Relative humidity is then calculated with the aid of:

(3) $$\mathrm{RH}=\left(\frac{x_{w}\cdot p}{p_{\mathrm{sv}}}\right)\times 100\%$$

The saturated vapor pressure of water is calculated from, for example, an Antoine relation [8]:

(4) $$p_{\mathrm{sv}}=133\cdot 10^{A-\frac{B}{C+T}}$$

Coefficients are *A* = 8.07131, *B* = 1730.63, *C* = 233.426, valid for 1–100 ° C, *T* in °C and *p_sv_* in *Pa*.

**Figure 1.**
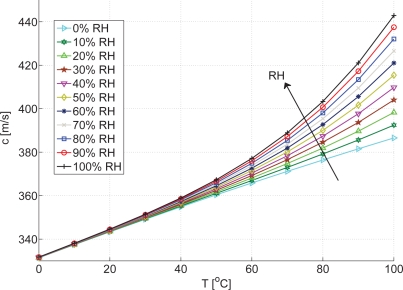
Speed of sound *vs.* temperature and relative humidity according to [2], *p* = 101.3 *kPa*, 314 *ppm CO*_2_.

The relation between the speed of sound, temperature and relative humidity according to Equation 2 to 4 is given in Figure 1. Note that sensitivity for *c* increases with increasing temperature and with increasing *RH*.

**Figure 2.**
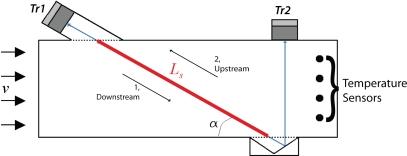
Schematic trajectories.

The speed of sound is determined by measuring the ultrasonic transit time of the acoustic signal on a trajectory. The transit time is influenced by the air-steam flow velocity, which is taken into account by averaging the speed of sound in upstream and downstream direction:

(5) $$\begin{array}{r} t_{m}=\frac{t_{1}+t_{2}}{2}\\ c=\frac{L_{t}}{t_{m}} \end{array}$$

with *t_m_* the transit time averaged in *s. L_t_* is the total length of the acoustic trajectory in *m* between transducers *Tr*_1_ and *Tr*_2_, see Figure 2. *t*_1_ is the transit time in downstream direction and *t*_2_ the transit time in upstream direction in *s*.

The average gas flow velocity is determined from the difference in transit time in upstream and downstream direction over the part of the acoustic trajectory *L_s_*. *L_s_* is the part of the acoustic trajectory where the ultrasonic waves have a component in the direction of the gas flow (thick outline in Figure 2). The average transit time is given by:

(6) $$t_{m}=\frac{t_{1}+t_{2}}{2}$$

At a part of the total acoustic trajectory, *L_t_*, the acoustic trajectory is perpendicular or outside the main flow. Gas flow velocity has no effect on the transit time here. This part of the trajectory is *L_d_*:

(7) $$L_{d}=L_{t}-L_{s}$$

The average transit time over trajectory *L_d_* is then given by:

(8) $$t_{d}=\frac{L_{d}}{L_{t}}t_{m}$$

Transit times in downstream and upstream direction over trajectory *L_s_* are:

(9) $$\begin{array}{c} t_{s1}=t_{1}-t_{d}\\ t_{s2}=t_{2}-t_{d} \end{array}$$

Due to superposition of the speed of sound on the gas flow velocity over trajectory *L_s_*, transit times in downstream and upstream direction are:

(10) $$\begin{array}{c} t_{s1}=\frac{L_{s}}{c+v\cdot \text{cos}\left(\alpha \right)}\\ t_{s2}=\frac{L_{s}}{c-v\cdot \text{cos}\left(\alpha \right)} \end{array}$$

with *α* the angle between flow direction and the acoustic trajectory *L_s_*, see Figure 2. Rearranging 10 and eliminating the speed of sound results in an average gas flow velocity of:

(11) $$v=\frac{L_{s}}{2\cdot \text{cos}\left(\alpha \right)}\frac{t_{s2}-t_{s1}}{t_{s1}\cdot t_{s2}}$$

Equation 11 allows determination of the average gas velocity from known dimensions (trajectory length and angle) and measured values (transit times) only, without the need of parameters of the gas which affect *c*.

### 2.1. Sensitivity and Accuracy

The main advantages of the acoustic humidity sensor become clear by observing the sensitivity of the relative humidity measurement on temperature. Relative humidity is determined by separate, but instantaneous, speed of sound and temperature measurements. Sensitivity of relative humidity is then given by:

(12) $$\begin{array}{r} \Delta \mathrm{RH}=\chi \Delta T\\ \chi ={\frac{\partial \mathrm{RH}}{\partial c}|}_{T=c}\cdot {\frac{\partial c}{\partial T}|}_{\mathrm{RH}=c} \end{array}$$

**Figure 3.**
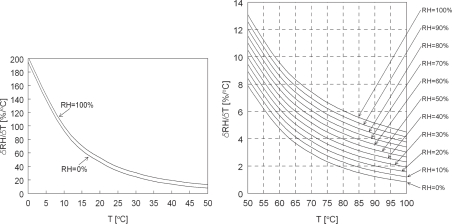
Derivative of relative humidity to temperature *vs.* temperature for relative humidity of 0–100 %. For example: at 90 °C and 20 %*RH,* an error of 1 °C in temperature induces a 2 % error in relative humidity.

The equation above is graphically represented by Figure 3. Relative humidity is very dependent on temperature. The accuracy of the relative humidity measurement is dominated by the accuracy of the independent temperature measurement.

In practice, accuracy is limited to the accuracy of reference relative humidity sensors at calibration. At temperatures below 50 °C, a small error in temperature results in large errors in humidity measurement. However, in the range of 50–100 °C very accurate humidity measurements over the full range of 0–100 %*RH* are possible, given a typical temperature measurement accuracy of ± 0.1 °C. This in contrast to other popular relative humidity measurement techniques like capacitive humidity sensors which become less accurate at high humidity and temperature levels [1], typically far worse than 2 %*RH* above 80 °C. Moreover, at constant temperature, variations in relative humidity can be measured very fast, at about 100 *Hz*, because the response time mainly depends on the speed of sound and typical transit times of the acoustic trajectory. Other popular relative humidity measurement techniques like capacitive humidity sensors suffer from response times in the order of seconds, depending on gas flow velocity. Although *CO*_2_-concentration and pressure also affect speed of sound, thus the relative humidity, these influences are negligible for *CO*_2_ in the *ppm* range and for pressures from 75 to 105 *kPa* [2].

## 3. Construction

**Figure 4.**
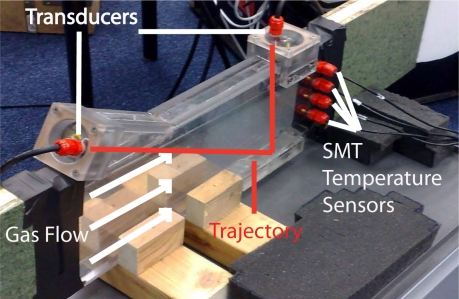
Overview.

An overview of the device without insulation is shown in Figure 4. The measurement section is constructed of PolyCarbonate plates of 10 *mm* thickness which form a rectangular duct with inner dimensions 18 × 130 *mm* and 500 *mm* length. Two ultrasonic transducers (operation at a frequency of 50 *kHz*) and four SMT temperature sensors are mounted in the duct as shown schematically in Figure 2. The temperature sensors are Smartec SMT 160-30 sensors in TO-18 housing. These sensors are chosen for their size, resolution (0.01 °C typically) and easy connection possibilities. The transducers and temperature sensors are connected to a programmable transmitter with calibration data. The transmitter communicates with Innovation Handling in-house developed software ClimaView for data acquisition on a PC. For preliminarily tests in a heat exchanger test rig, see 6.1, acquisition frequency is set to 1 *Hz*. Gas flow in the duct is measured at constant temperature and flow. In the heat exchanger test rig, fast response times are of minor importance.

## 4. Calibration

Temperature sensors are calibrated from 0 to 100 °C with an insulated Julabo MP open bath circulator and a reference thermometer. Accuracy of each SMT sensor is 0.14 °C. To take possible temperature gradients into account, temperatures are averaged over the height and weighed by the corresponding mass velocity [9]. Accuracy of the averaged temperature over four sensors is 0.07 °C. Details on calibration of the temperature sensors are given in [10].

The length of the acoustic trajectory is calibrated by measuring the transit times at no flow conditions for given temperature and relative humidity in an insulated reference box. *c* is known and transit times *t*_1_ and *t*_2_ should be equal. *L*_t_ is found to be 502.8 ± 0.1 *mm. L_s_* is determined by the design of the measurement device and is found to be 260.0 ± 0.1 *mm*.

The average gas flow velocity is calibrated over a range of 0 to 12 *m/s* to 0.13 *m/s* accurate in a wind tunnel with a reference flow meter [11].

**Figure 5.**
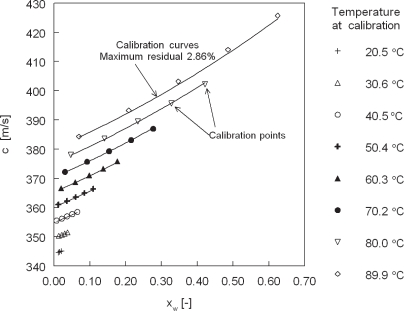
Speed of sound vs. water vapor mole fraction, calibration at various temperatures with the lines representing Equation 2 with constants given in Table 2.

Relative humidity measurements are calibrated in a Weiss SB22-300 climate chamber with a Michell S4000 cooled mirror optical dewpoint hygrometer, accurate to ±1 %*RH* and a psychrometer better than 3 %*RH* accurate. Calibration is performed at ambient pressure. A field of 40 measurements is assessed: temperatures from 20.0 to 90.0 °C in steps of 10.0 °C at relative humidities of 10 to 90 % in steps of 20 %. This results in coefficients of Equation 2 as given in Table 2. Comparison between the calibration points and the approximation by Equation 2 with the coefficients of Table 2 is shown in Figure 5.

## 5. Dynamic Response

One of the major advantages of acoustic sensors in general is the fast response time, because the response time mainly depends on the speed of sound and typical transit times of the acoustic trajectory. However, if the goal of the acoustic sensor is to measure relative humidity in a duct, a separate temperature measurement in the duct is needed. Response times are dependent on the slowest measurement, in this case the temperature sensors which have time constants of about 5 *s*. With temperature fluctuations in time, the relative humidity measurement will show a delay. Various methods to minimize this delay are considered:

1. Use smaller temperature sensors, with smaller time constants.
2. Damp the thermal fluctuations by installation of a large thermal mass at the duct inlet. This overcomes erroneous measurement of relative humidity due to temperature fluctuations, but cancels out the advantage of the fast response of the acoustics.
3. Install temperature sensors with different time constants and use the derivative of the fastest temperature sensor to correct the slowest temperature sensor.

These strategies are now under consideration to further improve the acoustic relative humidity sensor.

## 6. Results and Discussion

### 6.1. Preliminary Test in a Heat Exchanger Test Rig

**Figure 6.**
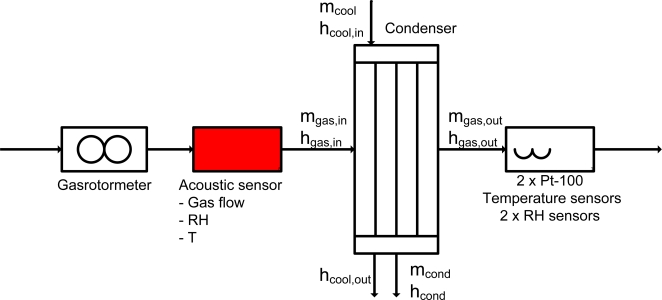
Test rig scheme.

The measurement device is preliminarily tested at the test section inlet of a wind tunnel for condensing heat exchangers at the Department of Mechanical Engineering, Eindhoven University of Technology, see [9]. A flow scheme is shown in Figure 6, with the definitions given below.

Heat flow rate from the gas is:

(13) $$Q_{\mathrm{gas}}={\dot{m}}_{\mathrm{gas},\mathrm{in}}\cdot h_{\mathrm{gas},\mathrm{in}}-{\dot{m}}_{\mathrm{gas},\mathrm{out}}\cdot h_{\mathrm{gas},\mathrm{out}}$$

with

(14) $${\dot{m}}_{\mathrm{gas},\mathrm{out}}={\dot{m}}_{\mathrm{gas},\mathrm{in}}-{\dot{m}}_{\mathrm{cond}}$$

Heat flow rate to the condensate is:

(15) $$Q_{\mathrm{cond}}={\dot{m}}_{\mathrm{cond}}\cdot h_{\mathrm{cond}}$$

and heat flow rate to the coolant:

(16) $$Q_{\mathrm{cool}}={\dot{m}}_{\mathrm{cool}}\cdot (h_{\mathrm{cool},\mathrm{out}}-h_{\mathrm{cool},\mathrm{in}})$$

The energy balance is given by:

(17) $$Q_{\mathrm{gas}}=Q_{\mathrm{cool}}+Q_{\mathrm{cond}}$$

with a deviation of:

(18) $$\mathrm{DEV}=\left|\frac{Q_{\mathrm{gas}}-\left(Q_{\mathrm{cool}}+Q_{\mathrm{cond}}\right)}{Q_{\mathrm{gas}}}\right|\cdot 100\%$$

**Figure 7.**
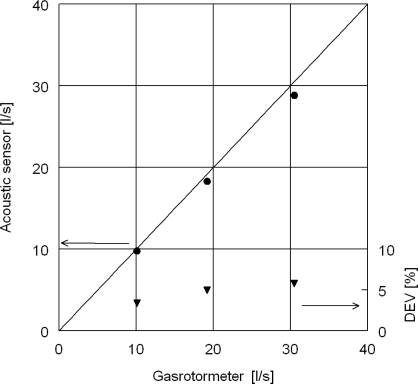
Volume flow rate of acoustic sensor calculated from average velocity in duct with *A* = 2.34 · 10^−3^ *m*^2^ *vs*. volume flow rate in a gasrotormeter. Corresponding mean velocities are 4 – 12 *m/s*. Each point is averaged over 200 *s*, *σ* < 0.5%.

The acoustic flow measurements are compared with the volumetric flow rate of dry air measured by a gasrotormeter positioned upstream the acoustic measurement device, see Figure 7. Deviation between the volume flow rate of the gasrotormeter and the calculated volume flow rate is 6% at worst. Note that the acoustic volume flow rate is calculated by straightforward multiplication of the average gas flow velocity with the flow area, without any compensation for flow profile, temperature gradients or nonlinearities [12,13]. Assessment of design improvements of acoustic flow rate measurements is given, for example, in [14] and is out of scope of the present study.

**Figure 8.**
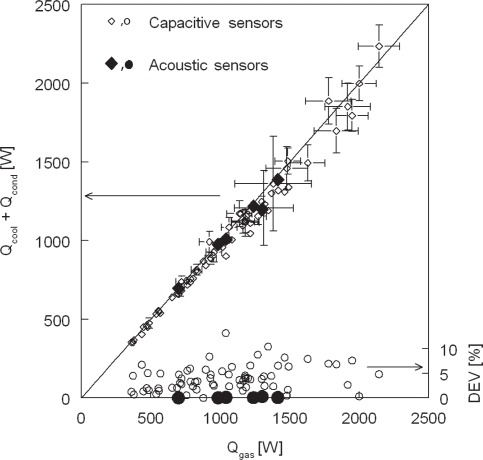
Energy balance measured in condensing steam-air flow with capacitive humidity sensors and acoustic humidity sensors.

Energy balance measurement accuracy improved by replacing capacitive humidity sensors at the gas flow inlet by the acoustic measurement device, see Figure 8. Gas inlet conditions varied from 20–40 %*RH* at 80 °C and 2–6 *m/s* for the points shown in Figure 8.

## 7. Conclusions

In this study, an in-line acoustic relative humidity sensor for air-steam mixtures in duct flow has been designed. The measurement device is capable of measuring line averaged gas velocity, temperature and humidity instantaneously by applying two ultrasonic transducers and an array of four SMT temperature sensors. Measurement range is gas velocity of 0–12 *m/s*, 0–100 °C and 0–100% relative humidity at ambient pressure. Main advantage over conventional humidity sensors is the high sensitivity at high *RH* at temperatures exceeding 50 °C, with accuracy increasing with increasing temperature. The sensors are non-intrusive and resist highly humid environments. Accuracy for line averaged flow velocity is 0.13 *m/s*, average temperature 0.07 °C after calibration. With this temperature measurement accuracy, intrinsic accuracy of relative humidity is better than 2 %*RH* above 50 °C and within 1 %*RH* from 70 to 100 °C. The practical accuracy in relative humidity at constant temperature solely depends on the the humidity calibration with the cooled mirror optical dewpoint hygrometer and a psychrometer, which is typically 1 to 3 %*RH* best practice, respectively [1]. Preliminary tests at the test section inlet of a wind tunnel for condensing heat exchangers have shown improved accuracy in the measurement of the energy balance.

## Figures and Tables

**Table 1.** Constituents of standard dry air.

Constituent	Molar mass *M_i_* [10^−3^*kg* · *mol*^−1^]	Mole fraction *x_i_*	Contribution *x_i_ M_i_* [10^−3^*kg* · *mol*^−1^]
*N*_2_	28.0134	0.78084	21.8739833
*O*_2_	31.9988	0.209476	6.7029806
*Ar*	39.948	0.00934	0.3731143
*CO*_2_	44.010	0.000314	0.0138191
*Ne*	20.183	18.18 · 10^−6^	0.0003669
*He*	4.0026	5.24 · 10^−6^	0.0000210
*CH*_4_	16.04303	2.0 · 10^−6^	0.0000321
*Kr*	83.8	1.14 · 10^−6^	0.0000955
*H*_2_	2.01594	0.5 · 10^−6^	0.0000010
*N*_2_*O*	44.0128	0.27 · 10^−6^	0.0000119
*CO*	28.01	0.19 · 10^−6^	0.0000053
*Xe*	131.3	0.087 · 10^−6^	0.0000114
*H*_2_*O*	18.01534	0.0	0.0

**Table 2.** Calibrated coefficients for Equation 2.

Coefficient	Calibrated value
*a*_0_	332.2424
*a*_1_	0.576691
*a*_2_	−0.000472
*a*_3_	47.597133
*a*_4_	0.1158039
*a*_5_	−0.000691
*a*_6_	−1.82 · 10^−7^
*a*_7_	3.73 · 10^−8^
*a*_8_	2.93 · 10^−10^
*a*_9_	−85.20931
*a*_10_	−0.228525
*a*_11_	5.91 · 10^−5^
*a*_12_	29.33397
*a*_13_	−2.15 · 10^−13^
*a*_14_	29.179762
*a*_15_	0.00483
*L_t_*	0.5026

## Nomenclature

**Table:**

*B*	Second virial coefficient	[*m*^3^*mol*^−1^]
*L*	Length	[*m*]
*M*	Molar mass	[*kg* · *mol*^−1^]
*Q*	Heat flow rate	[*W*]
*R*	Universal gas constant	[*J* · *mol*^−1^*K*^−1^]
*RH*	Relative Humidity	[%]
*T*	Temperature	[°C]
*a_i_*	Calibration coefficients	[−]
*c*	Speed of sound at measurement frequency	[*m* · *s*^−1^]
*c*_0_	Speed of sound (zero frequency)	[*m* · *s*^−1^]
*h*	Enthalpy	[*J* · *kg*^−1^*K*^−1^]
*ṁ*	Mass flow rate	[*kg* · *s*^−1^]
*p*	Pressure	[*Pa*]
*p_sv_*	Saturation pressure	[*Pa*]
*t*	Time	[*s*]
*v*	Velocity	[*m* · *s*^−1^]
*x_c_*	Mole fraction *CO*_2_	[*mole/mole mixture*]
*x_w_*	Mole fraction water	[*mole/mole mixture*]
*α*	Angle	[*°*]
*γ*	Specific heat ratio, $\frac{c_{p}}{c_{v}}$	[−]
*σ*	Standard deviation	[%]
*χ*	Error in relative humidity due to temperature error	[% ·*° C*^−1^]
